# ﻿Molecular and morphological evidence for a new species of *Pogostemon* (Lamiaceae) from Hainan Island, China

**DOI:** 10.3897/phytokeys.188.76611

**Published:** 2022-01-21

**Authors:** Langxing Yuan, Gan Tan, Wenhua Zhang, Bine Xue, Jiwen Deng, Lei Liu, Gang Yao

**Affiliations:** 1 Tropical Crops Genetic Resources Institute, Chinese Academy of Tropical Agricultural Sciences, Haikou 571101, China Tropical Crops Genetic Resources Institute, Chinese Academy of Tropical Agricultural Sciences Haikou China; 2 College of Forestry and Landscape Architecture, South China Agricultural University, Guangzhou 510642, China Haikou Duotan Wetlands Institute Haikou China; 3 Haikou Duotan Wetlands Institute, Haikou 570125, China South China Agricultural University Guangzhou China; 4 College of Horticulture and Landscape Architecture, Zhongkai University of Agriculture and Engineering, Guangzhou 510225, China Zhongkai University of Agriculture and Engineering Guangzhou China; 5 Yinggeling Branch of Hainan Tropical Rainforest National Park, Baisha 572800, China Yinggeling Branch of Hainan Tropical Rainforest National Park Baisha China

**Keywords:** China, Lamiaceae, *Pogostemon*, Pogostemoneae, taxonomy

## Abstract

*Pogostemonhainanensis*, a new species of Lamiaceae from Hainan Island, China, is described. The phylogenetic position of the new species within *Pogostemon* was investigated based on analyses of the nuclear ribosomal internal transcribed spacer (nrITS) and five plastid markers (viz. *matK*, *psbA-trnH*, *rbcL*, *rsp16*, *trnL-F*). The results show that *P.hainanensis* is supported to be a member of subgenus Pogostemon and is sister to *P.parviflorus*, a species widely distributed from Eastern Himalaya, through the Indo-China peninsula to China. Morphologically, the new species can be distinguished from all the other taxa of subgenus Pogostemon in having long petioles usually 4.5‒11.5 cm in length, and the calyx teeth 2/3 to subequal as long as the calyx tube. The new species differs from *P.parviflorus* further by its obviously double serrate leaf margin, spikes of inflorescence usually 2.5–8.0 cm long, calyx 4‒5 mm long and corolla 6–7 mm long.

## ﻿Introduction

*Pogostemon* Desf. is the largest genus in Pogostemoneae, Lamioideae, Lamiaceae (Zhao et al. 2021), and it is circumscribed recently in its broad sense that includes *Pogostemon* s.s. and *Dysophylla* Bl. (Yao et al. 2016; Zhao et al. 2021). The genus consists of more than 80 species distributed mainly in the tropical and subtropical Asia, and with five species endemic to Africa (Bhatti and Ingrouille 1997; Yao et al. 2015). The highest species diversity of the genus is found in the Indian subcontinent (Bhatti and Ingrouille 1997). Morphologically, the genus can be easily distinguished from other Pogostemoneae members by the presence of exserted stamens bearing moniliform hairs (Bhatti and Ingrouille 1997; Yao et al. 2015). Based on morphological and molecular evidence, the genus was divided into two subgenera, viz. subgenus Pogostemon and subgenus Dysophyllus (Bl.) Bhatti & Ingr. ex. G. Yao, Y.F. Deng & X.J. Ge (Yao et al. 2016). The former subgenus is characterized as perennial subshrubs, shrubs or rarely perennial herbs, spikes of inflorescence with more than two lateral branches, bracts and bracteoles large and usually broad-ovate, ovate or rarely lanceolate; while the latter subgenus is characterized as annual herbs or rarely perennial herbs or subshrubs, inflorescence a single terminal spike or rarely with two lateral branches, bracts and bracteoles small and narrow, and usually lanceolate, linear or filiform (Yao et al. 2016).

Based on results from extensive field investigations conducted recently, multiple new species of *Pogostemon* were reported, such as *P.nudus* Bongcheewin & Pramali from Thailand (Bongcheewin et al. 2017), *P.guamensis* Lorence & W.L. Wagner from Guam, Mariana Islands (Lorence et al. 2020), and *P.monticola* from Taiwan, China (Liu et al. 2021). In addition, the rare species *P.dielsianus* Dunn, which is endemic to southwestern China and known previously only from its type collected in 1905 (*G. Forrest 875*, E00087126, K000249619), was also rediscovered in a recent scientific field trip (Hu et al. 2021). Thus, extensive field investigations should be conducted and more new discoveries might be revealed, enabling better understanding of the biodiversity of the genus *Pogostemon* as well as for other biological groups.

In our taxonomic revision of Chinese *Pogostemon* (Yao et al. 2015), a specimen (*Z. Huang 36483*, IBSC-0585902) of *Pogostemon* collected in 1934 from Lingshui Hsien of Hainan Province, China, seemed to be very different from all the other congeneric taxa, especially in its large ratio of the length of calyx teeth and calyx tube (2/3 to ca. 1.0). However, due to the unavailability of intact leaves and flowers for measurements, the specimen was not further studied and the species that it represented was not included in our previous study (Yao et al. 2015). Recently, during a field investigation in Yinggeling Nature Reserve, Hainan Province, China, one of the authors (L.X. Yuan) collected a *Pogostemon* specimen that is very similar to the specimen *Z. Huang 36483* in plant morphology. Later, another two wild populations of the same species were discovered in Jiaxi Nature Reserve and Qixianling, Hainan Province, China. Detailed morphological comparison and specimen examination confirmed that the newly collected specimens are conspecific with *Z. Huang 36483* and the species is different from all the other *Pogostemon* taxa, thus it is formally described here. The phylogenetic position of the new species within *Pogostemon* is also studied here based on analyses of the nuclear ribosomal internal transcribed spacer (nrITS) and five plastid regions (*matK*, *psbA-trnH*, *rbcL*, *rps16*, *trnL-F*).

## ﻿Materials and methods

### ﻿Morphological study

Specimens of *Pogostemon* deposited in the herbaria BM, E, HITBC, IBK, IBSC, K, KUN, L, PE, US, NAS, TAI and NY were studied carefully in the present study. Herbarium abbreviations cited in the present study follow the Index Herbariorum (Thiers 2013 onwards). Extensive field investigations of Chinese *Pogostemon* were conducted over the last decade. Morphological characters of stems, leaves, inflorescences, flowers and nutlets were photographed and measured.

### ﻿Phylogenetic study

To study the phylogenetic position of the new species within the genus *Pogostemon*, a phylogenetic study of the genus was performed, based on analyses of six DNA markers (nrITS, *matK*, *psbA-trnH*, *rbcL*, *rps16*, *trnL-F*), following Yao et al. (2016). Total genomic DNA of the new species was extracted from silica gel-dried leaves (voucher specimens: *L.X. Yuan 20210206001* & *20210207001*; IBSC) using a Plant Genomic DNA Kit (Biomed, Shenzhen, China). Detailed information of primers of relevant DNA markers used in Polymerase Chain Reaction (PCR) amplification and sequencing, as well as the procedures of PCR, can be found in our previous study (Yao et al. 2016). All the DNA sequences used in Yao et al. (2016) and some *Pogostemon* sequences provided by other authors (Bendiksby et al. 2011; Hu et al. 2021) were downloaded from NCBI (www.ncbi.nlm.nih.gov). In total, 57 accessions representing 35 species of *Pogostemon* were sampled, in which 34 accessions representing 16 species of subgenus Pogostemon and 23 accessions representing 19 species of subgenus Dysophyllus were included. Other nine genera of Pogostemoneae (viz., *Achyrospermum* Bl., *Anisomeles* R. Br., *Colebrookea* Sm., *Comanthosphace* S. Moore, *Craniotome* Rchb., *Eurysolen* Prain, *Leucosceptrum* Sm., *Rostrinucula* Kudô., *Microtoena* Prain), the genus *Gomphostemma* Wall. ex Benth. of Gomphostemmateae and the genus *Colquhounia* Wall. of Colquhounieae were selected here as outgroups based on phylogenetic framework provided by Yao et al. (2016). Detailed information of all species sampled and sequences used are available in Appendix I.

All the DNA sequences were aligned using MAFFT 7.221 (Katoh and Standley 2013), and then three different datasets were constructed: the cpDNA dataset (including *matK*, *psbA-trnH*, *rbcL*, *rps16*, *trnL-F*), the nrITS dataset, and the combined dataset (including all the six DNA markers). All the three datasets were analyzed using two approaches: Bayesian Inference (BI) and Maximum Likelihood (ML) were conducted using MrBayes v. 3.2.7a (Ronquist and Huelsenbeck 2003) and RAxML (Stamatakis 2006) on the CIPRES cluster (Miller et al. 2010), respectively. The models of nucleotide substitution of the six DNA markers used were selected independently under the Akaike Information Criterion (AIC) using jModeTest v. 3.7 (Posada 2008): GTR+I+Γ for nrITS, GTR+Γ for *matK*, TrN+Γ for *psbA-trnH*, GTR+I for *rbcL*, TVM+Γ for *rps16* and GTR+I for *trnL-F*. Detailed methods for BI and ML analyses could refer to the phylogenetic study conducted in Yao et al. (2021), except that each of Markov Chain Monte Carlo (MCMC) analysis was run for 10,000,000 generations and sampling every 500 generations in BI analysis. Number of generations in BI analysis was sufficient, because the effective sample size (ESS) of all parameters were over 200 as evaluated in Tracer v. 1.6 (Rambaut et al. 2014), and the average standard deviations (SD) of split frequencies for the dataset was below 0.01. The first 25% of the trees obtained in BI analysis were discarded as burn-in and then posterior probabilities (PP) were determined from the posterior distribution. A rapid bootstrap (BS) analysis using the model GTR+Γ with 1000 pseudoreplicates was conducted to obtain the support values in ML analysis.

## ﻿Results

### ﻿Phylogenetic analyses

**Figure 1.**
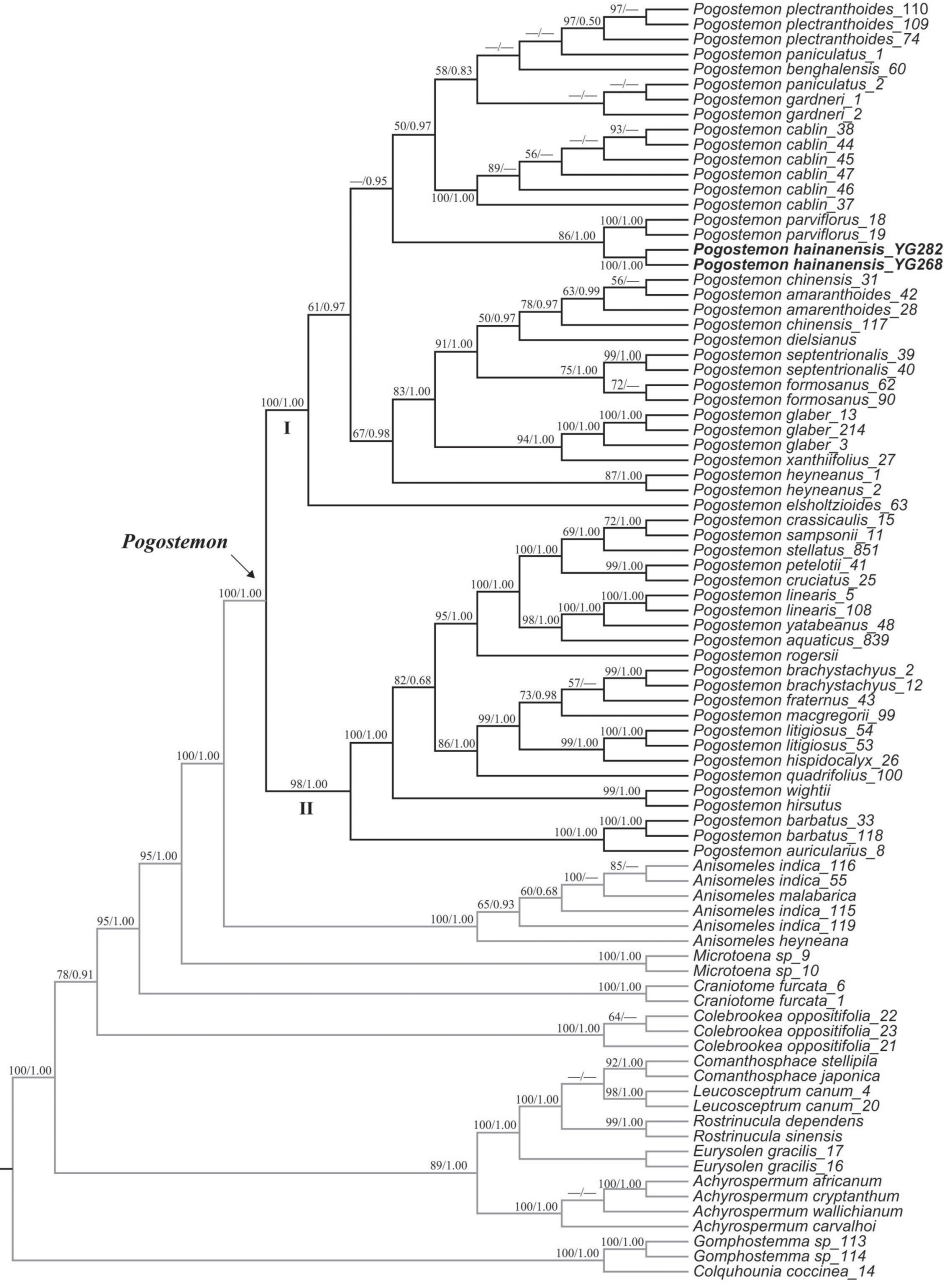
Maximum likelihood (ML) tree of *Pogostemon* and its relatives inferred from the combined dataset (including nrITS, *matK*, *psbA-trnH*, *rbcL*, *rps16*, *trnL-F*). Bootstrap (BS) value in ML analysis and posterior probility (PP) in Bayesian inference (BI) is indicated on the left and right of slanting bar associated with phylogenetic node, respectively. Dashes denote that the phylogenetic node associated was not supported or the BS value is < 50% in ML anlaysis or PP < 0.50 in BI. The crown node of *Pogostemon* is show by the arrowhead.

The cpDNA dataset, nrITS dataset and combined dataset alignments contained 3872 bp, 707 bp and 4,579 bp, respectively. The topology of *Pogostemon* and its relatives derived from the nrITS dataset was largely consistent with that derived from the cpDNA dataset, except several nodes that were lowly supported (defined here as BS < 80% or PP < 0.80) (Suppl. material 1 and 2). Phylogenetic relationships derived from the combined dataset (Figure 1) were much better resolved compared with those obtained from both the cpDNA dataset and nrITS dataset (Suppl. material 1 and 2), thus we focus on describing phylogenetic relationships based on the result derived from the combined dataset.

Results from analyses of the combined dataset recovered a highly supported (defined here as BS ≥ 90% or PP ≥ 0.99) sister relationship between the two genera *Anisomeles* and *Pogostemon* (BS = 100%, PP = 1.00). Phylogenetic relationships of the genus *Pogostemon* obtained here are also consistent with those reported in Yao et al. (2016). In *Pogostemon*, two major clades were highly supported: Clade I representing the subgenus Pogostemon (BS = 100%, PP = 1.00) and clade II representing the subgenus Dysophyllus (BS = 98%, PP = 1.00) (Fig. 1). The monophyly of the new species is well-supported (BS = 100%, PP = 1.00) and it is nested deeply within the first clade. Furthermore, a sister relationship between the new species and *P.parviflorus* Benth. is moderately supported (defined here as 80% ≤ BS < 90% or 0.80 ≤ PP < 0.99) in ML analysis (BS = 86%) and highly supported in BI analysis (PP = 1.00). Detailed information about the phylogenetic relationships of other nodes can be referred to Figure 1.

### ﻿Morphological comparison

**Figure 2.**
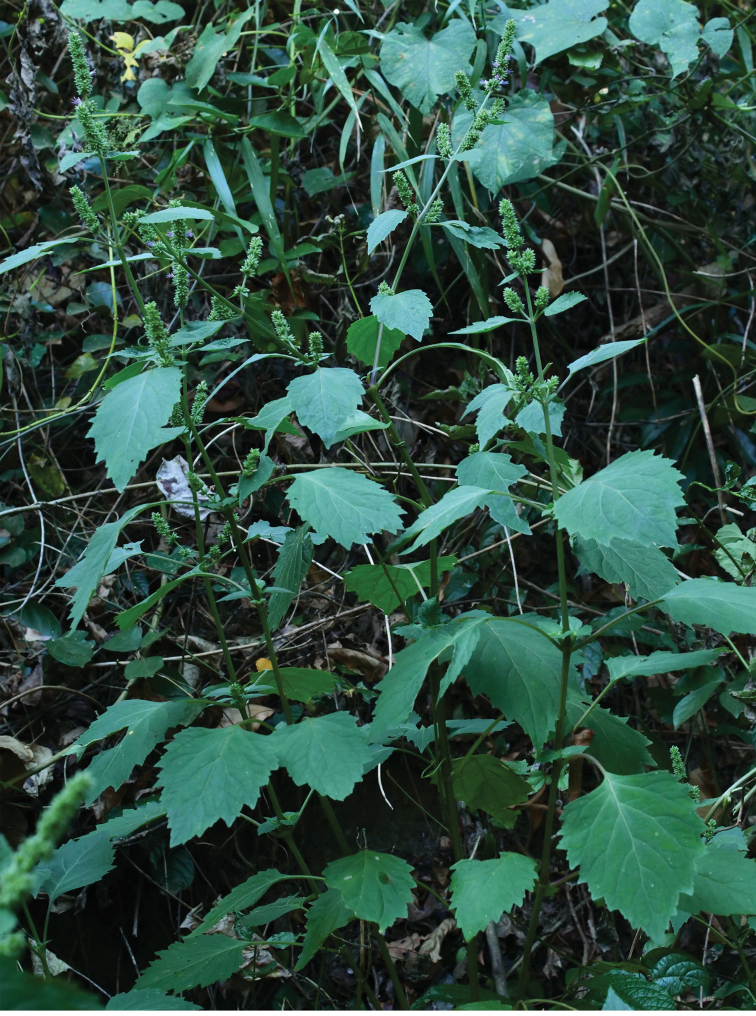
Habit of *Pogostemonhainanensis*.

**Figure 3.**
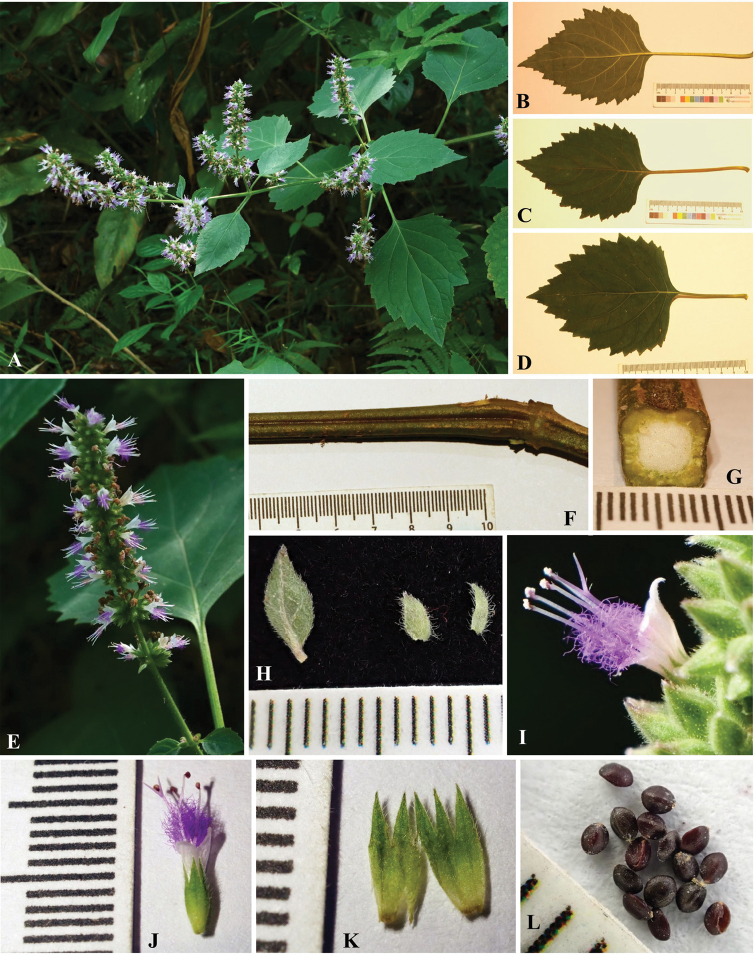
*Pogostemonhainanensis***A** branch **B** leaf **C, D** leaf **E** spike of inflorescence **F** stem **G** cross section of stem **H** bract (the left one) and bracteoles (the right two) **I, J** flower **K** calyx **L** nutlets.

A detailed morphological comparison between the new species and other species of *Pogostemon* was conducted. A series of morphological characters of the new species, such as the obviously double serrated leaf margin (Figs 2, 3A–D), long petioles (usually 4.5‒11.5 cm long; Fig. 3B–D), and the large ratio of the length of calyx teeth and calyx tube (2/3–1.0; Fig. 3J‒K), can be used to distinguish the new species from all the other members of subgenus Pogostemon easily. In habit, the new species is similar to *P.parviflorus* Benth. and *P.septentrionalis* C.Y. Wu & Y.C. Huang, which also have wild populations discovered in South China (Yao et al. 2015). However, the new species further differs from *P.parviflorus* in having large spikes of inflorescence (usually 2.5‒8 cm long and 9‒12 mm wide; Fig. 3A, E), larger calyx (4‒5 mm long; Figure 3J) and corolla (6‒7 mm long), besides above-mentioned three traits. In contrast, *P.parviflorus* has obscurely or shallowly double crenated to double serrated leaves margin, shorter petioles (1‒4.5 cm long), smaller spikes of inflorescence (1‒4.5 cm long and 8‒10 mm wide), calyx (4‒4.2 mm long) and corolla (4‒4.5 mm long), as well as the ratio of the length of calyx teeth and calyx tube (less than 1/2) (Yao et al. 2015). While *P.septentrionalis* has shorter petioles (0.5‒5.5cm long), narrow spikes of inflorescence (7‒9 mm in diameter), smaller calyx (3‒4 mm long) and corolla (4‒4.5 mm long), the ratio of the length of calyx teeth and calyx tube (1/3‒1/2), and larger nutlets (0.9‒1.0 mm long) (Yao et al. 2015).

Only two species of subgenus Pogostemon [viz., *P.cablin* (Blanco) Benth. and *P.esquirolii* (H. Léveillé) C. Y. Wu & Y. C. Huang] were recorded previously in Hainan Island, China (Guangdong Institute of Botany 1977; Wu and Huang 1977). While the specific name *P.esquirolii* had been reduced previously to be a synonym of *P.glaber* Bentham by Rehder (1935), and this treatment was accepted by Yao et al. (2015) in their taxonomic revision of Chinese *Pogostemon*. The new species can be distinguished from *P.cablin* (a cultivated species in China) (Yao et al. 2015) by a series of morphological traits, such as its spikes of inflorescences are 9‒12 mm in diameter (Fig. 3A, E) (vs. 13‒18 mm in diameter), calyx 4‒5 mm long (Fig. 3J‒K) (vs. 6‒8 mm long), the ratio of the length of calyx teeth and calyx tube is 2/3‒1.0 (Figure 3J‒K) (vs. ca. 1/4). While the new species differs from *P.glaber* by its leaves margin obviously double serrate (Figs 2, 3A‒D) (vs. usually shallowly double serrate or double crenate), spikes of inflorescences usually 2.5–8.0 cm long (Fig. 3A, E) (vs. 3.0–15.0 cm long), calyx 4‒5 mm long (Figure 3J‒K) (vs. 3‒4.5 mm long), the ratio of the length of calyx teeth and calyx tube is 2/3‒1.0 (Fig. 3J‒K) (vs. ca. 1/3), corolla 6–7 mm long (vs. 3–5.5 mm long).

A detailed morphological comparison among the new species and its relatives or morphologically similar species also can be referred to Table 1.

**Table 1.** Morphological comparison among *Pogostemonglaber* Bentham, *P.hainanensis* L.X. Yuan & Gang Yao, *P.parviflorus* Bentham and *P.septentrionalis* Wu & Huang.

Morphology	*P.glaber*	*P.hainanensis*	*P.parviflorus*	*P.septentrionalis*
Leaf blade	Margin shallowly double serrate or double crenate	Margin obviously double serrate	Margin obscurely double crenate to double serrate	Margin double serrate
Petiole	Usually 3–5 cm long	Usually 4.5‒11.5 cm long	Usually 1–4.5 cm long	Usually 0.5–5.5 cm long
Inflorescence	Usually 3.0–15.0 cm long and 6‒10 mm wide	Usually 2.5–8.0 cm long and 9‒12 mm wide	Usually 0.7–3.5 cm long and 8–10 mm wide	Usually 3–13 cm long and 7–9 mm wide
Calyx	3‒4.5 mm long; the ratio of the length of calyx teeth and calyx tube is ca. 1/3	4‒5 mm long; the ratio of the length of calyx teeth and calyx tube is 2/3‒1.0	4‒4.2 mm long; the ratio of the length of calyx teeth and calyx tube is less than 1/2	3–4 mm long; the ratio of the length of calyx teeth and calyx tube is 1/3–1/2
Corolla	3–5.5 mm long	6–7 mm long	4‒4.5 mm long	4–4.5 mm long

## ﻿Discussion

Morphologically, the new species has spikes of inflorescence with more than two later branches (Figs 2, 3A), bracts and bracteoles large and broad-ovate, ovate or lanceolate in shape (Fig. 3H), indicating that the species is a member of subgenus Pogostemon, which is further confirmed in phylogenetic analyses (Figure 1). According to current circumscription, about 28 species (including the new species) are accepted in subgenus Pogostemon (Bhatti and Ingrouille 1997; Yao et al. 2015; Liu et al. 2021), among which 16 species were sampled in the present phylogenetic analyses (Figure 1). The species *Pogostemonmonticola* T.C. Hsu, S.W. Chung, S.H. Liu & W.J. Huang described recently from Taiwan, China, was not sampled in the present phylogenetic study due to the unavailability of DNA material or DNA sequences, but its phylogenetic position within subgenus Pogostemon was resolved and it was closely related to *P.formosanus* Oliver and *P.septentrionalis* Wu & Huang (Liu et al. 2021). While for the other 11 species of subgenus Pogostemon not sampled in the present phylogenetic analyses, viz. *P.cristatus* Hassk., *P.griffithii* Prain, *P.hispidus* Prain, *P.latifolius* (C.Y. Wu & Y.C. Huang) Gang Yao, *P.nelsonii* Doan, *P.nepetoides* Stapf, *P.pubescens* Benth., *P.purpurascens* Dalzell, *P.tuberculosus* Benth., *P.villosus* Benth., and *P.wattii* C.B. Clarke, they also have a series of morphological characters that can be distinguished from the new species from Hainan, China, especially in terms of the margin of leaves, the length of petioles, the size of calyx and the ratio of the length of calyx teeth and calyx tube. Detailed information about the morphological characters of these 11 species can be referred in Bhatti and Ingrouille (1997) and Yao et al. (2015). Thus, as mentioned above, the combined evidence from morphological and phylogenetic analyses well supported the independently taxonomic status of the new species in *Pogostemon*.

### ﻿Taxonomic treatment

#### ﻿ Pogostemon hainanensis

Taxon classificationPlantaeLamialesLamiaceae

L.X. Yuan & Gang Yao sp. nov.

D83A7BA7-CC45-54F9-AA07-E10BD9F63BB3

urn:lsid:ipni.org:names:77248974-1

Figures 2 , 3

##### Diagnosis.

The species is similar to *Pogostemonparviflorus* Benth. in general morphology, but differs from the latter by its leaves margin obviously double serrate, petioles usually 4.5‒11.5 cm long, spikes of inflorescence up to 8 cm long, calyx 4‒5 mm long, corolla 6–7 mm long, and the calyx teeth is 2/3 to subequal as long as the calyx tube.

##### Type.

China. Hainan province, Yinggeling Nature Reserve, Nanleshan, Fanyang, Wuzhishan, 18°54'38.25"N, 109°22'26.58"E, at an elevation of about 570 m, 6 February 2021, *L.X. Yuan 20210206001* (holotype, IBSC; isotypes: IBSC, KUN).

##### Description.

Perennial herbs or shrubs, 0.8–2 m tall. Stem erect, 6–8 mm in diameter, 4-angular, slightly dilated at nodes, a few branched, strigose, or villous at the upper part. Leaves opposite; blade ovate, rarely ovate-lanceolate, (5.5–) 9–13.5 × (2.5) 6.5–10.5 cm, papery or membranous, strigose on both surfaces, base broadly cuneate, margin obviously double serrate, entire at base, apex acuminate; midvein elevated abaxially, lateral vein 3–4 (rarely 5) pairs on each side of the midvein, slightly elevated abaxially; petioles (1.5–) 4–11.5 cm long, ca. 1 mm in diameter, strigose. Spikes of inflorescence (1.0–) 2.5–8.0(–10.5) cm long, 9–12 mm in diameter, terminal and axillary, interrupted basally in long spikes, usually with more than two lateral branches; peduncle (1–) 2–4 cm long, densely villous; verticillasters many-flowered, flowers sessile. Bracts oblong, 5–13 × 2.5–5 mm, strigose, midvein elevated abaxially, lateral vein 1–2 pairs on each side of the midvein or sometimes obscure; bracteoles ovate-lanceolate to narrowly lanceolate, 2.5–5 × 0.7–1.8 mm, strigose. Calyx tubular-inflated, 4–5 mm long, 5-veined, strigose and sparsely golden glandular outside, sometimes sparsely strigillose inside at the upper part of tube; teeth 5, narrowly triangular, equal, 1.8–2 mm long, 0.6–0.8 mm wide at base, 2/3 to subequal as long as the calyx tube, subglabours or strigillose inside. Corolla white, 6–7 mm long, exserted evidently from calyx, 2-lipped, upper lip 3-lobed, lower lip entire. Stamens 4, erect, much exserted from corolla; filaments 7–7.5 mm long, all inserted at a height of ca. 2 mm in the tube, bearded at middle, bearded portion exserted; anther 1-locular, cell apex dehiscent. Style 7–7.5 mm long; stigma bifid, lobes subequal, 1.2–1.7 mm long. Disc ca. 0.6 mm long. Nutlets 4, ca. 0.7 × ca. 0.6 mm long, ellipsoid or slightly depressed globose, abaxially slightly flat, adaxially ribbed, black or dark brown.

##### Etymology.

*Pogostemonhainanensis* is named after its type locality, Hainan province, China.

##### Phenology.

Flowering from December to the next February and fruiting from January to April.

##### Paratype.

China. Hainan Province, Lingshui Hsien, 12 January 1934, near river, *Z. Huang 36483* (IBSC-0585902!); Ledong Hsien, Jiaxi Nature Reserve, 18°52'35.85"N, 109°10'36.99"E, at an elevation of about 800 m, 7 February 2021, *L.X. Yuan 20210207001* (IBSC); Baoting Hsien, near the Tiantan waterfall, 18°42'16.17"N, 109°41'50.55"E, at an elevation of about 560 m, 25 April 2021, *L.X. Yuan 20210425001* (IBSC).

##### Distribution and habit.

**Figure 4.**
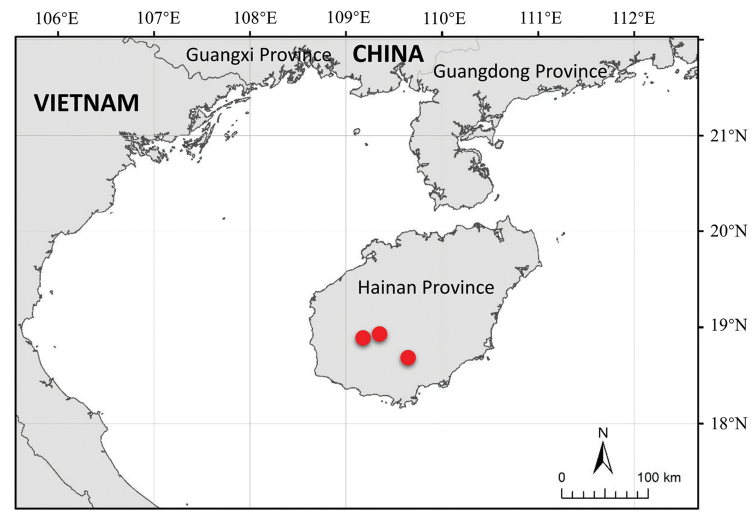
Distribution of *Pogostemonhainanense* (red circular).

The new species is endemic to Hainan Province, China (Figure 4). It grows under forests, usually near ravines, at an elevation of 550–800 m.

##### Chinese name.

Hai Nan Ci Rui Cao (海南刺蕊草).

## Supplementary Material

XML Treatment for Pogostemon hainanensis

## ﻿Appendix I

Sequences and taxa information for all samples used in the present study (–, missing data; *, newly-generated sequences). Accession numbers are given for nrITS, *matK*, *rbcL*, *rps16*, *trnH-psbA*, *trnL-F* (c-f or c-d/e-f; the characters “c”, “d”, “e” and “f” indicate different primers used for PCR and sequencing of the marker *trnL-F*, referred from Yao et al. 2016).

***Pogostemonamaranthoides*** Benth._28: –, KR608424, KR608489, KR608613, KR608548, KR608676; ***P.amaranthoides***_42: KR608745, KR608425, KR608490, KR608614, KR608549, KR608677; ***P.aquaticus*** (C. H. Wright) Press_839: KR608767, KR608468, KR608527, KR608655, KR608592, KR608717; ***P.auricularius*** (L.) Hassk._8: KR608761, KR608451, KR608513, KR608638, KR608575, KR608700; ***P.barbatus*** Bhatti & Ingr._33: KR608762, KR608452, KR608514, KR608639, KR608576, KR608701; ***P.barbatus***_118: KR608763, KR608453, KR608515, KR608640, KR608577, KR608702; ***P.benghalensis*** (Burm. f.) Kuntze: –, KR608444, –, HQ911592, KR608568, HQ911663/HQ911731; ***P.brachystachyus*** Benth._2: KR608775, KR608455, KR608517, KR608642, KR608579, KR608704; ***P.brachystachyus***_12: KR608774, KR608454, KR608516, KR608641, KR608578, KR608703; ***P.cablin*** (Blanco) Benth._37: KR608757, KR608438, KR608503, KR608627, KR608562, KR608690; ***P.cablin*** _38: KR608752, KR608439, KR608504, KR608628, KR608563, KR608691; ***P.cablin***_44: KR608753, KR608440, KR608505, KR608629, KR608564, KR608692; ***P.cablin***_45: KR608754, KR608441, KR608506, KR608630, KR608565, KR608693; ***P.cablin***_46: KR608755, KR608442, KR608507, KR608631, KR608566, KR608694; ***P.cablin***_47: KR608756, KR608443, KR608508, KR608632, KR608567, KR608695; ***P.chinensis*** C. Y. Wu & Y. C.Huang_31: KR608743, KR608426, KR608491, KR608615, KR608550, KR608678; ***P.chinensis***_117: KR608742, KR608449, KR608512, KR608637, KR608573, KR608699; ***P.crassicaulis*** (Benth.) Press_15: KR608770, KR608469, KR608528, KR608656, KR608593, KR608718; ***P.cruciatus*** (Benth.) Kuntze_25: KR608771, KR608466, KR608525, KR608653, KR608590, KR608715; ***P.dielsianus*** Dunn: MW194872, –, MW194874, MW194875, MW194873, MW194876; ***P.elsholtzioides*** Benth._63: –, KR608445, –, KR608633, KR608569, KR608720; ***P.formosanus*** Oliver_62: KR608744, KR608434, KR608499, KR608623, KR608558, KR608686; ***P.formosanus***_90: KR608779, KR608435, KR608500, KR608624, KR608559, KR608687; ***P.fraternus*** Miq.: KR608781, KR608461, –, KR608648, KR608585, KR608710; ***P.gardner*** Hook. f._1: MF303612, MF303632, –, –, –, –; ***P.gardner***_2: MF303603, MF303622, –, –, –, –; ***P.glaber*** Benth._3: KR608740, KR608431, KR608496, KR608620, KR608555, KR608683; ***P.glaber***_13: KR608739, KR608429, KR608494, KR608618, KR608553, KR608681; ***P.glaber***_214: KR608741, KR608430, KR608495, KR608619, KR608554, KR608682; ***P.hainanensis*** L.X. Yuan & Gang Yao_YG268: OL625022, OL616075, OL616077, OL616079, OL616081, OL616083; ***P.hainanensis***_YG282: OL625023, OL616076, OL616078, OL616080, OL616082, OL616084; ***P.heyneanus*** Benth._1: KR608751, KR608427, KR608492, KR608616, KR608551, KR608679; ***P.heyneanus***_2: –, HQ911401, –, FJ854069, –, FJ854297/FJ854184; ***P.hirsutus*** Benth.: –, HQ911397, –, FJ854070, –, FJ854298/FJ854185; ***P.hispidocalyx*** C. Y. Wu & Y. C. Huang_26: KR608780, KR608457, –, KR608644, KR608581, KR608706; ***P.linearis*** (Benth.) Kuntze_5: KR608764, KR608462, KR608521, KR608649, KR608586, KR608711; ***P.linearis***_108: KR608765, KR608463, KR608522, KR608650, KR608587, KR608712; ***P.litigiosus*** Doan ex Suddee & A. J. Paton_53: KR608776, KR608458, KR608519, KR608645, KR608582, KR608707; ***P.litigiosus***_54: KR608777, KR608459, KR608520, KR608646, KR608583, KR608708; ***P.macgregorii*** W. W. Sm._99: KR608778, –, –, –, –, –; ***P.paniculatus*** (Willd.) Benth._1: –, KR608450, –, –, KR608574, KR608721; ***P.paniculatus***_2: –, HQ911399, –, FJ854071, –, FJ854299/FJ854186; ***P.parviflorus*** Benth._18: KR608749, KR608436, KR608501, KR608625, KR608560, KR608688; ***P.parviflorus***_19: KR608750, KR608437, KR608502, KR608626, KR608561, KR608689; ***P.petelotii*** Doan ex G. Yao, Y. F. Deng & X. J. Ge_41: KR608772, KR608470, KR608529, KR608657, KR608594, KR608719; ***P.plectranthoides*** Desf._74: KR608760, KR608446, KR608509, KR608634, KR608570, KR608696; ***P.plectranthoides***_109: KR608758, KR608447, KR608510, KR608635, KR608571, KR608697; ***P.plectranthoides***_110: KR608759, KR608448, KR608511, KR608636, KR608572, KR608698; ***P.quadrifolius*** (Benth.) F. Muell._100: KR608773, KR608456, KR608518, KR608643, KR608580, KR608705; ***P.rogersii*** N E. Br.: KR608782, KR608460, –, KR608647, KR608584, KR608709; ***P.sampsonii*** (Hance) Press_11: KR608769, KR608465, KR608524, KR608652, KR608589, KR608714; ***P.septentrionalis*** C. Y. Wu & Y. C. Huang_39: KR608747, KR608432, KR608497, KR608621, KR608556, KR608684; ***P.septentrionalis***_40: KR608748, KR608433, KR608498, KR608622, KR608557, KR608685; ***P.stellatus*** (Lour.) Kuntze_851: KR608768, KR608464, KR608523, KR608651, KR608588, KR608713; ***P.wightii*** Benth.: MF303601, MF303620, –, –, –, –; ***P.xanthiifolius*** C. Y. Wu & Y. C. Huang_27: KR608746, KR608428, KR608493, KR608617, KR608552, KR608680; ***P.yatabeanus*** (Makino) Press_48: KR608766, KR608467, KR608526, KR608654, KR608591, KR608716;

**﻿Outgroups:*****Achyrospermumafricanum*** Hook. f. ex Baker: –, HQ911418, –, FJ853999, –, FJ854246/FJ854133; ***A.carvalhoi*** Gürke: –, HQ911412, –, FJ854001, –, FJ854248/FJ854135; ***A.cryptanthum*** Baker: –, HQ911415, –, FJ854002, –, FJ854249/FJ854136; ***A.wallichianum*** (Benth.) Benth. ex Hook. f.: –, –, –, HQ911594, –, HQ911666/HQ911734; ***Anisomelesheyneana*** Benth.: –, HQ911394, –, HQ911589, –, HQ911659/HQ911727; ***A.indica*** (L.) Kuntze_55: KR608726, KR608406, KR608471, KR608595, KR608530, KR608658; ***A.indica***_115: –, KR608407, KR608472, KR608596, KR608531, KR608659; ***A.indica***_116: KR608727, KR608408, KR608473, KR608597, KR608532, KR608660; ***A.indica***_119: KR608728, KR608409, KR608474, KR608598, KR608533, KR608661; ***A.malabarica*** (L.) R. Br. ex Sims: –, HQ911396, –, FJ854013, –, FJ854260/FJ854147; ***Colebrookeaoppositifolia*** Sm._21: KR608732, KR608414, KR608479, KR608603, KR608538, KR608666; ***C.oppositifolia***_22: KR608733, KR608415, KR608480, KR608604, KR608539, KR608667; ***C.oppositifolia***_23: KR608734, KR608416, KR608481, KR608605, KR608540, KR608668; ***Comanthosphacejaponica*** (Miq.) S. Moore: –, HQ911407, –, FJ854029, –, FJ854272/FJ854159; ***C.stellipila*** S. Moore: –, HQ911408, –, FJ854030, –, FJ854273/FJ854160; ***Craniotomefurcate*** (Link) Kuntze_1: KR608730, KR608412, KR608477, KR608601, KR608536, KR608664; ***C.furcate***_6: KR608731, KR608413, KR608478, KR608602, KR608537, KR608665; ***Eurysolengracilis*** Prain_16: KR608735, KR608417, KR608482, KR608606, KR608541, KR608669; ***E.gracilis***_17: KR608736, KR608418, KR608483, KR608607, KR608542, KR608670; ***Leucosceptrumcanum*** Sm._4: KR608738, KR608419, KR608484, KR608608, KR608543, KR608671; ***L.canum***_20: KR608737, KR608420, KR608485, KR608609, KR608544, KR608672; ***Microtoena sp.***_9: KR608729, KR608410, KR608475, KR608599, KR608534, KR608662; ***M.> sp.***_10: –, KR608411, KR608476, KR608600, KR608535, KR608663; ***Rostrinuculadependens*** (Rehder) Kudô: –, HQ911405, –, FJ854074, –, FJ854302/FJ854189; ***R.sinensis*** (Hemsl.) C. Y. Wu: –, HQ911406, –, FJ854075, –, FJ854303/FJ854190; ***Colquhouniacoccinea*** Wall._14: KR608722, KR608421, KR608486, KR608610, KR608545, KR608673; ***Gomphostemma sp.***_113: KR608723, KR608422, KR608487, KR608611, KR608546, KR608674; ***G. sp.***_114: KR608724, KR608423, KR608488, KR608612, KR608547, KR608675.

## Supplementary materials

Supplementary material 1 Figure S1 Data type Phylogenetic tree Explanation note Supplementary Figure 1 Maximum likelihood (ML) tree of *Pogostemon* and its relatives inferred from the cpDNA dateset (including matK, psbA-trnH, rbcL, rps16, trnL-F). https://binary.pensoft.net/file/636519This dataset is made available under the Open Database License (http://opendatacommons.org/licenses/odbl/1.0/). The Open Database License (ODbL) is a license agreement intended to allow users to freely share, modify, and use this Dataset while maintaining this same freedom for others, provided that the original source and author(s) are credited.Langxing Yuan, Gan Tan, Wenhua Zhang, Bine Xue, Jiwen Deng, Lei Liu, Gang Yao

Supplementary material 2 Figure S2 Data type Phylogenetic tree Explanation note Supplementary Figure 2 Maximum likelihood (ML) tree of *Pogostemon* and its relatives inferred from the nrlTS dataset. https://binary.pensoft.net/file/636520This dataset is made available under the Open Database License (http://opendatacommons.org/licenses/odbl/1.0/). The Open Database License (ODbL) is a license agreement intended to allow users to freely share, modify, and use this Dataset while maintaining this same freedom for others, provided that the original source and author(s) are credited.Langxing Yuan, Gan Tan, Wenhua Zhang, Bine Xue, Jiwen Deng, Lei Liu, Gang Yao
