# Supplementary figures and images for: ﻿Molecular and morphological evidence for a new species of Pogostemon (Lamiaceae) from Hainan Island, China
Source: PhytoKeys. 2022 Jan 21;188:177–91. doi: 10.3897/phytokeys.188.76611 (PMC8799628; doi:10.3897/phytokeys.188.76611)

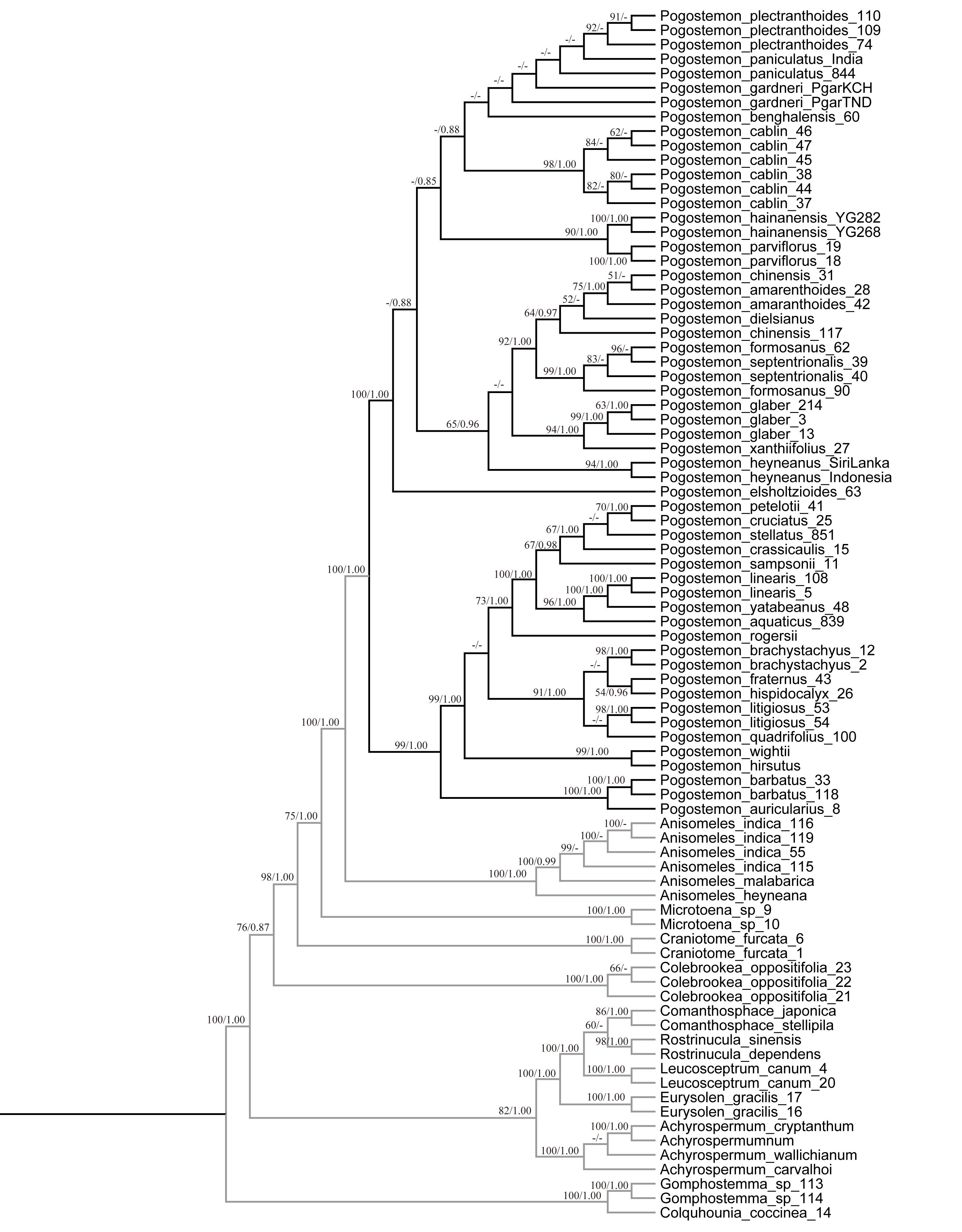

Supplement: Supplementary material 1 — Figure S1 [file phytokeys-188-177-s001.jpg]

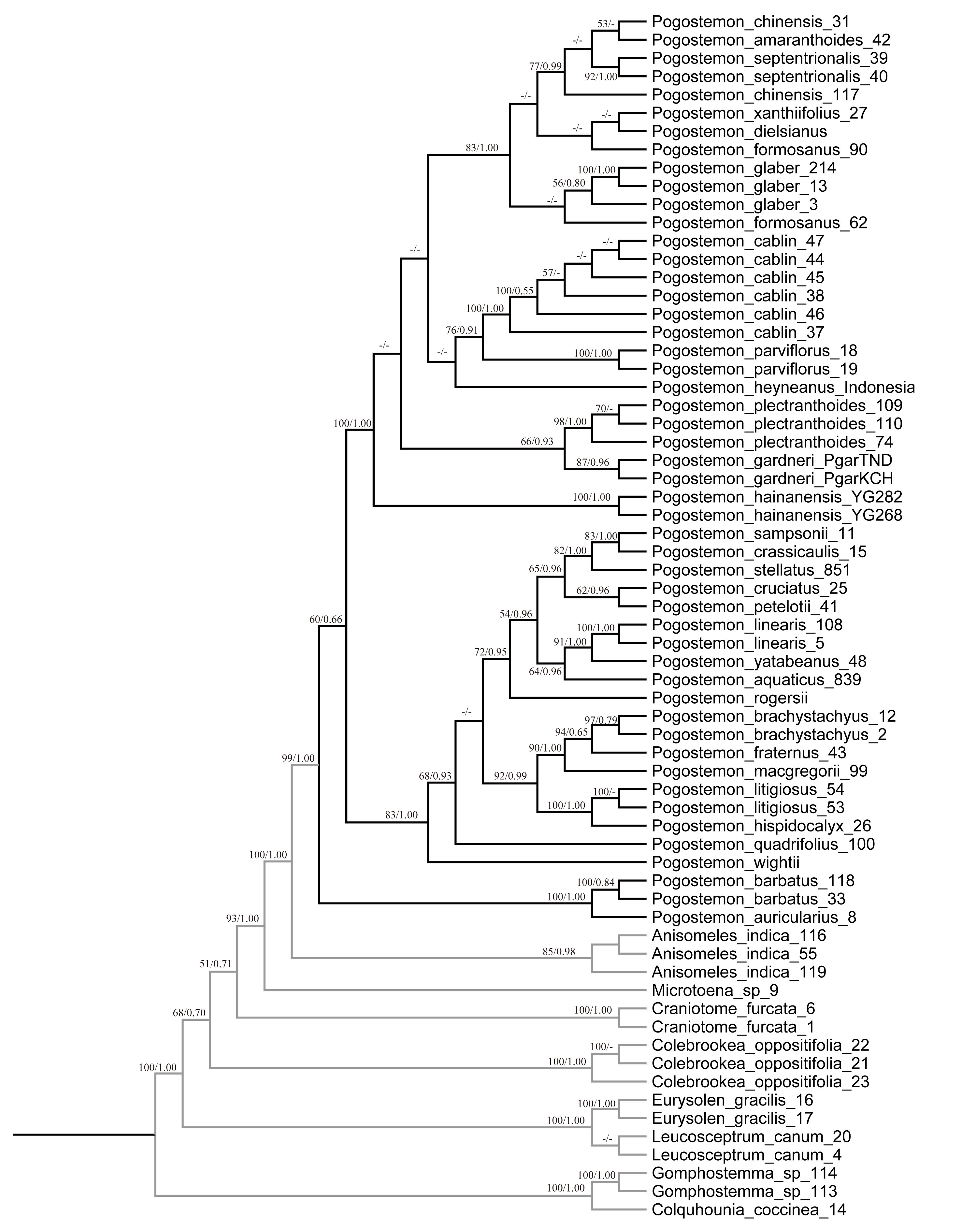

Supplement: Supplementary material 2 — Figure S2 [file phytokeys-188-177-s002.jpg]
